# HyperBeta: characterizing the structural dynamics of proteins and self-assembling peptides

**DOI:** 10.1038/s41598-021-87087-0

**Published:** 2021-04-08

**Authors:** Marco S. Nobile, Federico Fontana, Luca Manzoni, Paolo Cazzaniga, Giancarlo Mauri, Gloria A. A. Saracino, Daniela Besozzi, Fabrizio Gelain

**Affiliations:** 1 Department of Industrial Engineering and Innovation Sciences, Eindhoven University of Technology, Eindhoven, The Netherlands; 2 Department of Informatics, Systems and Communication, University of Milano-Bicocca, Milan, Italy; 3 SYSBIO/ISBE.IT Centre of Systems Biology, Milan, Italy; 4 Bicocca Bioinformatics, Biostatistics and Bioimaging Centre (B4), Monza, Italy; 5 Fondazione IRCCS Casa Sollievo della Sofferenza, Unità Ingegneria Tissutale, Viale Cappuccini 1, San Giovanni Rotondo, 71013 Foggia, Italy; 6 Department of Mathematics and Geosciences, University of Trieste, Trieste, Italy; 7 Department of Human and Social Sciences, University of Bergamo, Bergamo, Italy; 8 Center for Nanomedicine and Tissue Engineering (CNTE), A.S.S.T. Grande Ospedale Metropolitano Niguarda, Piazza dell’Ospedale Maggiore 3, 20162 Milan, Italy

**Keywords:** Computational biology and bioinformatics, Structural biology

## Abstract

Self-assembling processes are ubiquitous phenomena that drive the organization and the hierarchical formation of complex molecular systems. The investigation of assembling dynamics, emerging from the interactions among biomolecules like amino-acids and polypeptides, is fundamental to determine how a mixture of simple objects can yield a complex structure at the nano-scale level. In this paper we present HyperBeta, a novel open-source software that exploits an innovative algorithm based on hyper-graphs to efficiently identify and graphically represent the dynamics of $\beta$-sheets formation. Differently from the existing tools, HyperBeta directly manipulates data generated by means of coarse-grained molecular dynamics simulation tools (GROMACS), performed using the MARTINI force field. Coarse-grained molecular structures are visualized using HyperBeta ’s proprietary real-time high-quality 3D engine, which provides a plethora of analysis tools and statistical information, controlled by means of an intuitive event-based graphical user interface. The high-quality renderer relies on a variety of visual cues to improve the readability and interpretability of distance and depth relationships between peptides. We show that HyperBeta is able to track the $\beta$-sheets formation in coarse-grained molecular dynamics simulations, and provides a completely new and efficient mean for the investigation of the kinetics of these nano-structures. HyperBeta will therefore facilitate biotechnological and medical research where these structural elements play a crucial role, such as the development of novel high-performance biomaterials in tissue engineering, or a better comprehension of the molecular mechanisms at the basis of complex pathologies like Alzheimer’s disease.

## Introduction

Supra-molecular self-assembly arises from the interplay of non-covalent inter-molecular and intra-molecular interactions, ruling the autonomous organization of molecules into ordered patterns upon exposure to specific environmental conditions or external stimuli. Unlike covalent bonds, non-covalent interactions (electrostatic interactions, $\pi$-effects, van der Waals forces, hydrophobic effects) do not involve sharing of electron pairs between atoms. Hence, they are characterized by low energies, poor directionality, and reversibility^1^. These atomic-to-nanoscale features give rise to different macroscale properties such as self-healing or recovery of the original shape, usually not available to covalently bonded structures^2,3^. Most of the processes in biological systems—such as the formation of organelles, DNA replication or protein folding—emerge from spontaneous biomacromolecular self-assembly^3^. Inspired by these mechanisms, researchers have developed different classes of self-assembling molecules suitable for application in several fields, such as electronics^4^, material science^5^, and regenerative medicine^3^.

In the last decade, the rapid progresses in this field have been supported by the improvement of experimental and, in particular, computational methods. Among them, molecular dynamics (MD) played a major role in the investigation of supra-molecular self-assembly^6^. Conventional all-atom (AA) MD simulations usually consider atoms as the interaction sites, that is, the points where the potential energy functions of each interaction are calculated. However, due to their exceptional computational requirements, AA models can be inadequate for the investigation of complex systems along timescales that are comparable to reality. In order to overcome this intrinsic limitation of AA modeling, coarse-grained (CG) models have been developed^7^.

CG modeling consists in grouping multiple atoms as individual interaction sites, named grains or beads, thus reducing the overall computational effort. This strategy was widely applied to investigate various self-assembling systems, like peptides or lipids^8,9^. Two different approaches to CG modeling for biological systems have been proposed: (1) shape-based CG methods, where a small number of CG beads—typically, 10–50 beads with 200–500 atoms per bead— mimic the overall macro-molecule shape; (2) residue-based CG, where several atoms—typically, 10–20 atoms per bead—are grouped into a single CG interaction site, which usually represents a single residue, a side-chain, or a group of backbone atoms^9,10^. MARTINI is the best-known and wide-spread residue-based CG force field for the simulation of bio-molecular systems^9^. MARTINI CG-MD simulations are largely used in the field of supra-molecular chemistry and structural biochemistry. Despite their unquestionable advantages, i.e. reduced computational costs and accurate description of molecular movements, MARTINI CG-MD simulations suffer from a huge limitation in tracking the structural changes involved in protein folding. Indeed, these simulations do not allow to monitor the non-covalent interactions leading to the formation of secondary structures. In addition, in MARTINI CG-MD simulations the secondary structure arrangements are limited by imposing of harmonic potentials among backbone grains. This means that MARTINI CG-MD simulations are not suitable for the study of transitions among different secondary structures. Instead, MARTINI CG-MD simulations find applications for the study of the arrangements of protein structures within supramolecular aggregates, such as self-assembling peptides (SAPs) nano-fibrils. Due to the lack of information concerning non-covalent interactions, the main limitation of MARTINI CG simulation is that analytic tools for the quantitative tracking and visualization of self-assembling patterns are still lacking. The development of such tools is mandatory to achieve a deeper understanding of self-assembling phenomena^10,11^.

In supra-molecular chemistry, SAPs were widely used as models for the investigation of Alzheimer’s disease, and in the latter years they found several applications also in the field of tissue engineering^12^. SAPs self-assemble into $\alpha$-helix or $\beta$-sheet secondary structure patterns. The amount of $\beta$-sheet content in SAPs supra-molecular structures usually well-correlates to their mechanical properties at the macroscale, which, in turn, can have significant effects on either attached or encapsulated cells in tissue engineering applications^11^. In this work, we present HyperBeta , a novel tool that fills the gap in state-of-the-art methods for the analysis of CG molecular structures. HyperBeta was specifically developed for the analysis of CG-MD simulations of SAPs systems, and allows the automatic identification and real-time rendering of $\beta$-sheets in MARTINI CG-MD dynamics. Differently from Morphoscanner^13^, a tool for CG-MD simulations of SAPs systems, HyperBeta relies on an approach based on hyper-graphs and additional geometric constraints. Moreover, HyperBeta calculates several statistics about the composition of $\beta$-sheets, and embeds a high-quality 3D engine that exploits sophisticated visual cues to simplify the interpretation of distance and depth relationships among the grains and the peptides. The methodology presented in this paper was validated on multiple proteinaceous structures, showing that it can be successfully exploited to obtain relevant details on SAP processes and kinetics.

## Results

In the MD simulation analysis, the secondary structure assignment relies on the recognition of the hydrogen bond pattern or on the equivalent three-dimensional topological pattern of backbone atom groups. The features of MARTINI force-field hamper the analysis of CG-MD simulations with software like DSSP^14^ or STRIDE^15^: on the one hand, DSSP algorithm assigns secondary structures to single amino acid by identifying hydrogen bonds, which are not defined in MARTINI CG model; on the other hand, STRIDE assigns amino acid secondary structures by using hydrogen bonds and intra-chain dihedral angle potentials. The HyperBeta analysis workflow does not use the information inherent to molecular connectivity and intra-chain dihedral angles, whereas it relies on the CG beads Cartesian coordinates.

**Figure 1:**
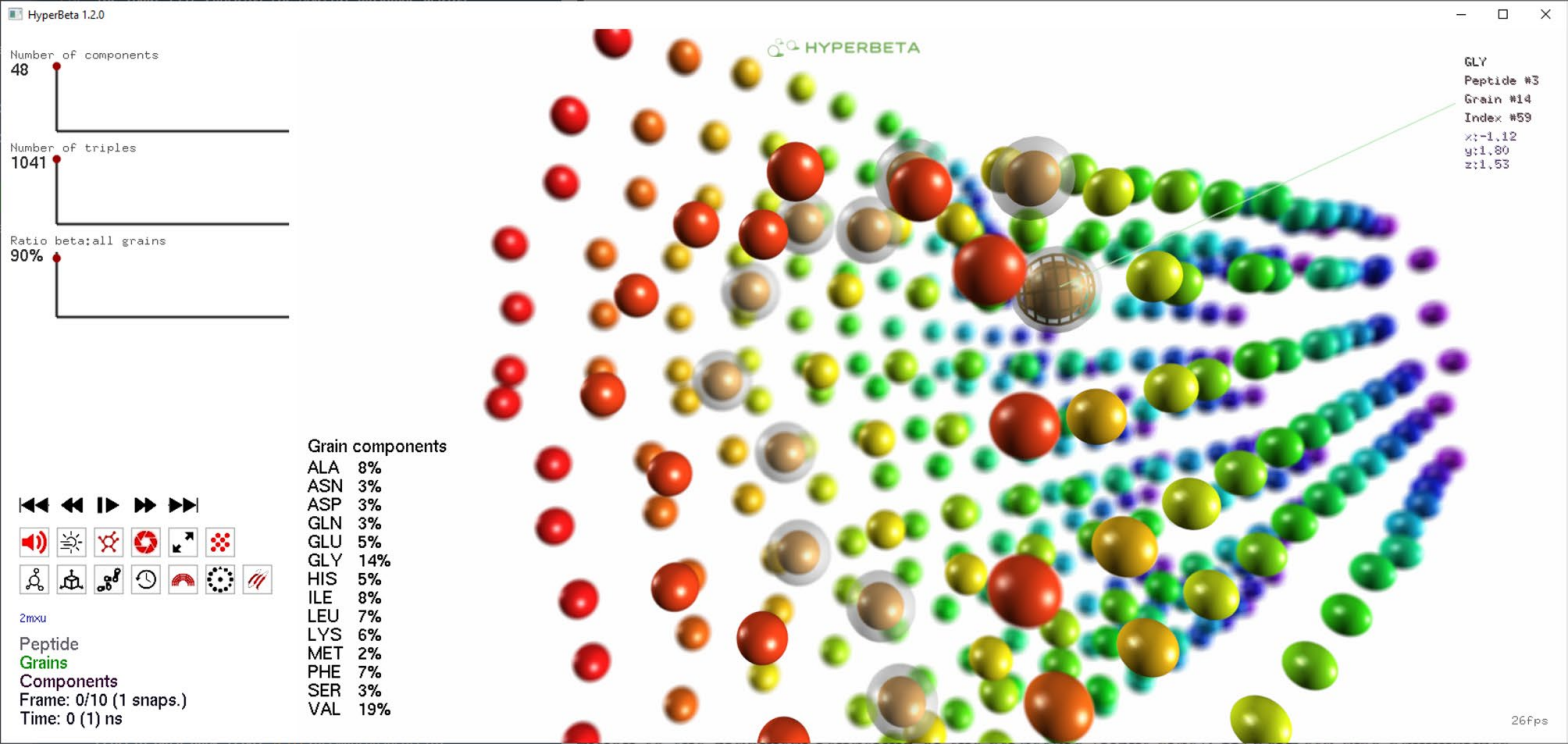
Screenshot of HyperBeta ’s graphical user interface. The grains of the structure are shown as spheres. Grains belonging to the same peptide are represented with the same color. Peptides can be selected by clicking on any grain; when selected, all grains in that peptide are shown as translucent spheres. To facilitate the interpretation of the spatial relationship between grains, simulated depth-of-field blurring is performed. In addition to the graphical rendering of the structure (right side), HyperBeta also summarizes the statistics about the identified $\beta$-sheets and the grain components involved in the structures (left side).

HyperBeta processes GROMACS files^16^ exported using the Gromos87 format, representing a single structure or multiple frames of a MD run, along with the number of amino-acid residues per peptide (group length, GL). The input file contains the MARTINI backbone grains corresponding to a single structure or multiple frames (here named “snapshots”) of a MD run. The user can easily introduce both information using HyperBeta ’s GUI (see Supplementary File #1). Once the GROMACS files are processed, HyperBeta ’s visualization tool visualizes the whole animation of the peptides self-assembly. As shown in Fig. 1, HyperBeta ’s rendering allows to track different peptides using different colors. When a grain or a peptide is selected, simulated fogging and depth-of-field^17^ provide visual cues of distance relationships between the objects. In addition, HyperBeta highlights the key statistics such as the number of components and their relative compositions in order to have a quantitative description of $\beta$-sheets. Then, HyperBeta summarizes other useful statistics such as the fraction of grains belonging to $\beta$-sheets in a translucent panel placed in the top-left corner of the screen.

In order to validate HyperBeta, we processed four protein structures with known characteristics downloaded from the Protein Data Bank (PDB)^18^. Then, the proteins were CG-mapped according to the MARTINI model and subsequently analyzed using HyperBeta. The tested structures were:

- the laminin-g-like module (PDB ID 1d2s), a high-molecular weight protein belonging to the extra-cellular matrix and constituting the biologically active part of the basal lamina ($GL=10$)^19^;
- the $A \beta (1-42)$ fibrils (PDB ID 2mxu), the initial and predominant constituents of the amyloid plaques that characterize Alzheimer’s disease ($GL=32$)^20^;
- an engineered *Boriella* OspA structure (PDB ID 2fkg) consisting of $\beta$-hairpin repeats connected by turn motifs ($GL=9$)^21^;
- the *Escherichia coli* $\beta$-clamp (PDB ID 3bep), a sub-unit of the DNA polymerase III holoenzime, characterized by antiparallel $\beta$-sheet structures ($GL=6$)^22^.

**Figure 2:**
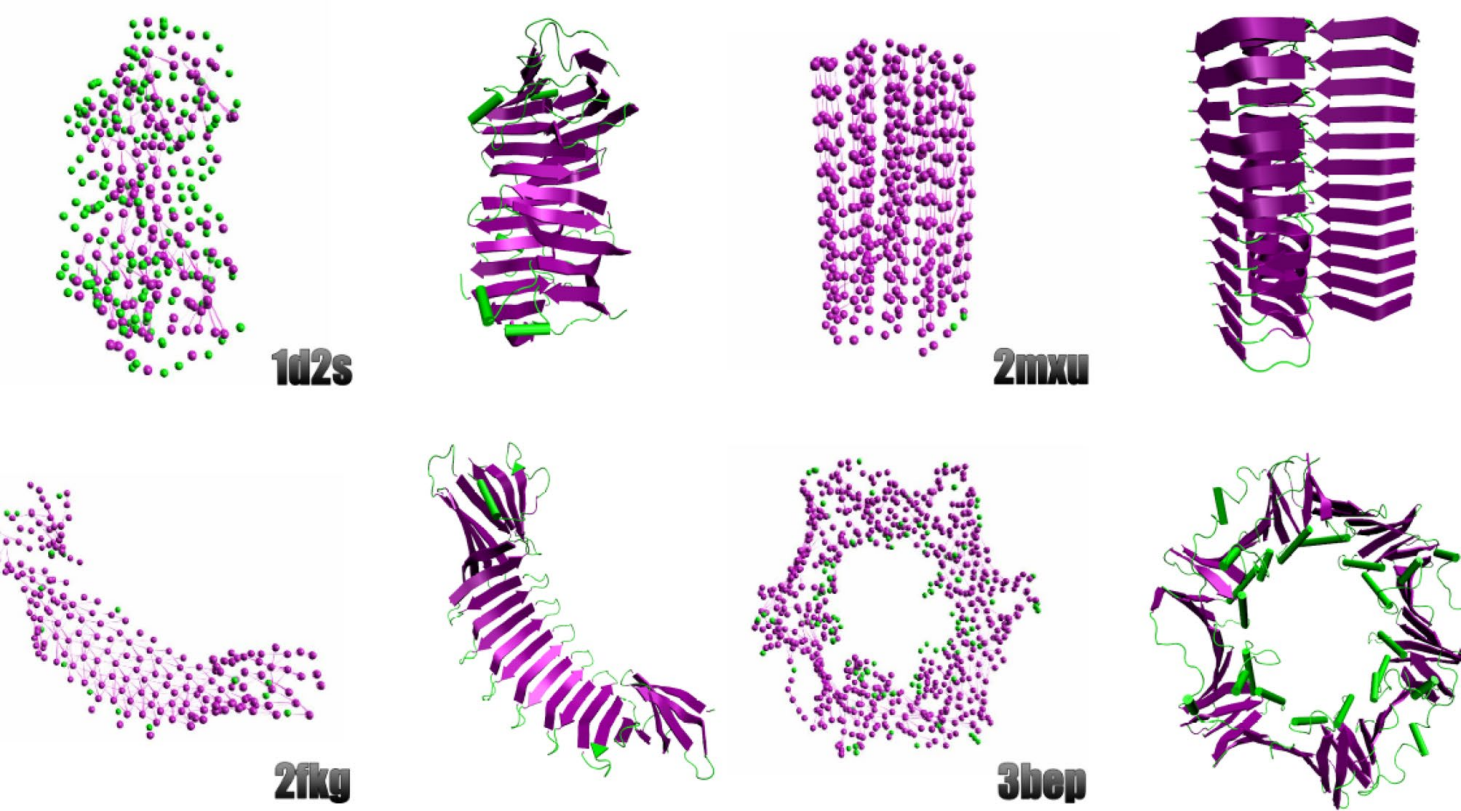
Validation of HyperBeta on different protein structures. Four PDB structures were analyzed with STRIDE and then compared to HyperBeta. The structures are (clockwise): the Laminin G-like domain (PDB ID: 1d2s); 42-residue $\beta$-amyloid fibril (PDB ID: 2mxu); the *E. coli* $\beta$-clamp (PDB ID: 3bep); engineered OspA (PDB ID: 2fkg). In each pair, the $\beta$-sheets (coarsened by the CG model) calculated by HyperBeta are shown on the left, while the corresponding united-atom structures rendered by VMD are shown on the right.

Figure 2 shows the result of this preliminary validation phase, presenting a comparison of the output produced by HyperBeta and rendered by HyperBeta ’s visualization tool by setting the angular threshold $\alpha = 0.89$ and the distance threshold $\varepsilon = 0.7$ nm (see Supplementary File #1 for further information and for comparison with Morphoscanner^13^), against the structures identified by STRIDE^23^, a well-established tool for secondary structure assignment, and rendered with VMD^24^. The putative $\beta$-sheets identified through HyperBeta in CG models correspond to the $\beta$-sheets identified in united-atom (UA) models through STRIDE and rendered with VMD.

Successively, five different SAPs MD trajectories were analyzed using HyperBeta with the same settings for $\alpha$ and $\varepsilon$. These trajectories were previously analyzed by Saracino et al.^13^. These systems comprised a total of identical 100 peptides for BMHP1-derived SAP sequences (B26: Btn-GGGPFASTKT , GL = 10; B24: Btn-GGGAFASTKT, GL = 10; 30: WGGGAFASTKT, GL = 10) and (LDLK)$_3$ SAP (GL = 12), and 50 plus 50 opposite charged peptides for the complementary assembling peptides (CAPs), whose sequences are (LDLD)$_3$ and (LKLK)$_3$ (GL = 12).

**Figure 3:**
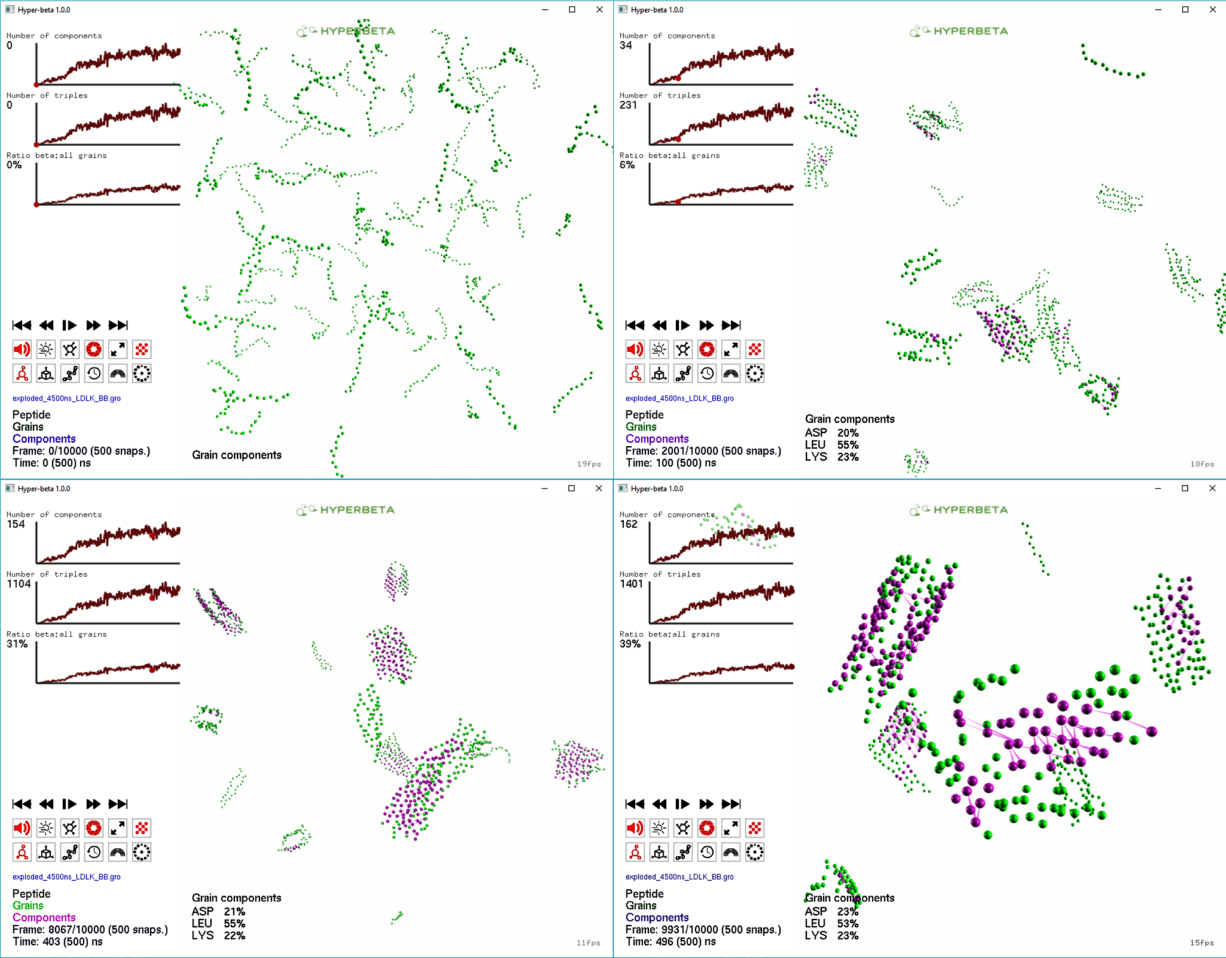
Analysis of (LDLK)$_3$ CG-MD trajectory simulation. Grain components refer to the type of grains belonging to the different $\beta$-sheets. The composition of the components remains constant, resulting from the alignment of Aspartic acid, Lysine and Leucine residues.

Figure 3 shows that, after 500 ns (from top to bottom, left to right), the (LDLK)$_3$ SAPs organize themselves into multiple independent cross-$\beta$ fibril seeds, which subsequently assemble into a “patchwork”-like aggregate^13^. Despite the huge variations of the number of triplets and components over time, the ratio between the different backbone grain types involved in the formation of the triplets remains stable. After the first 100 ns, the ratio between the numbers of Aspartic acid and Lysine backbone grains involved in the formation of the triplets is approximately equal to 1, as can be derived from the statistics at the left bottom corner of each panel. Indeed, the grain components of the triplets arise from the alternating alignment of opposite charge groups of Lysine and Aspartic Acid residues, resulting in $\beta$-sheet rich aggregates. As a matter of fact, as shown by Saracino et al.^13^, SAPs organization trend can be predicted by analyzing the first 500 ns of CG-MD simulation trajectories.

**Figure 4:**
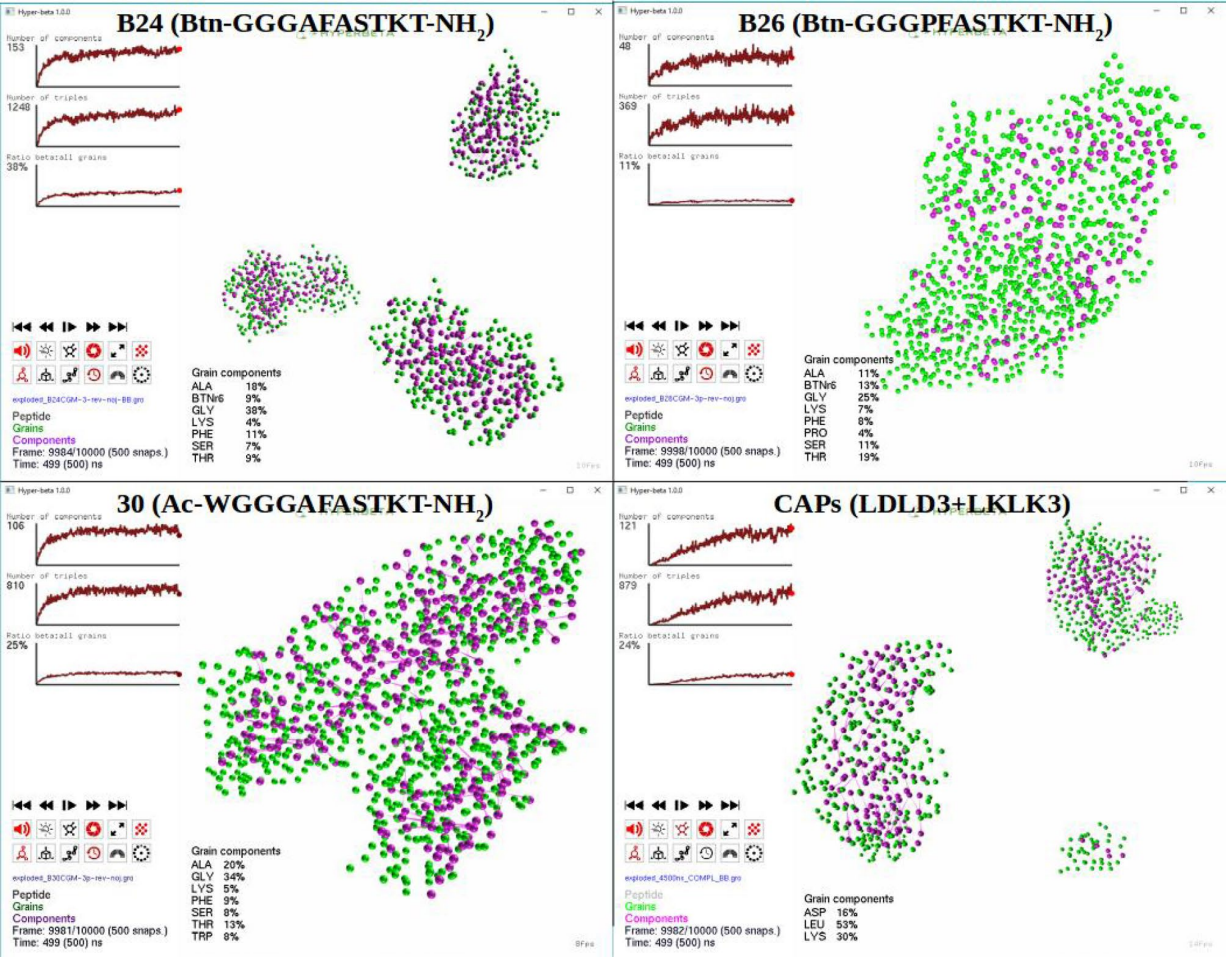
Supra-molecular organization of SAPs through HyperBeta with $\alpha = 0.89$ and $\varepsilon = 0.7$ nm. Three BMHP1-derived SAPs and CAPs simulations are analysed. B24 shows the highest number of grain components and the highest number of grains involved in the formation of triplets. The composition of the components is heterogeneous and the components mainly include hydrophobic residues such as Alanine, Glycine and Phenilalanine. B26 show the lowest number of components and the lowest number of triplets. These features are ascribable to the $\beta$-breaker effect of Proline. SAPs 30 assemble into a less structured aggregate, compared to B24. Such difference is mainly due to the presence of Tryptophan, instead of Biotin, at the N terminus position. CAPs assemble into well organized aggregates and the ratio between Lysine and Aspartic Acid in the components is approximately equal to 2.

As depicted in Fig. 4, SAP B24, the most promising sequence of the BMHP1-derived SAPs for neural tissue engineering applications, shows the highest number of components and triplets. Large part of these triplets consists of hydrophobic grains such as Alanine, Glycine, Phenilalanine. The Biotin grains form a highly dynamic and unstable network of putative $\beta$-sheets^13^. On the contrary, B26 shows the lowest number of components and triplets. This tendency is ascribable to the $\beta$-breaker effect of Pro residues and their favourable interactions with Biotin grains, which hampers the formation of stable $\beta$-sheets. These are in turn related to the amphipathic features of each moieties of Biotin, such as hydrophilicity of the ureido ring and hydrophobicity of thiophene ring and valeryl chain. Despite the high similarity with SAP B24, SAPs 30 assemble into a less structured aggregate. Such difference is ascribable to the substitution of Biotin with the aromatic amino-acid Tryptophan, at the N terminus position. As depicted in Fig. 4, CAPs assemble into stable aggregates, such as (LDLK)$_3$ SAPs. The ratio between the number of Lysine and Aspartic acid backbone grains, which are involved in the formation of triplets, is approximately equal to 2. Such feature is ascribable to the alternating arrangement of CAPs within bi-layered aggregates. Indeed, each (LDLD)$_3$ peptide is paired with two neighboring (LKLK)$_3$ peptides.

## Discussion

HyperBeta is a novel software for the analysis and rendering of MARTINI CG-MD structures developed to monitor secondary structure patterns, obtained as a consequence of the establishment of non-covalent interactions between grains. The rationale is that in MARTINI CG-MD simulations, the assignment of secondary structure patterns relies on the recognition of the three-dimensional topological pattern of backbone atom groups. More in details, in MARTINI models, hydrogen bonds are implicitly described through the definition of particular bead types, such as N$_{da}$ type^10,11^, and protein secondary structures are constrained through the introduction of harmonic potentials among backbone grains. The harmonic potentials force the peptide chains to adopt extended conformations. Thus, in MARTINI CG-MD simulations, peptide self-assembly may result into $\beta$-sheet rich aggregates, whose geometries resemble those of a distorted lattice.

HyperBeta was also designed to provide a pleasant and productive user experience, by exploiting frustum culling, backface culling, and level-of-detail balancing to improve the reactivity of the real-time rendering even in the case of massive structures. Differently from any existing visualization tool, HyperBeta performs advanced visual cues like simulated depth-of-field to improve the interpretation of distance and depth relationships^17^ between grains and peptides. Although HyperBeta was developed to investigate the relationships between grains and $\beta$-structures, it also provides the possibility of rendering $\beta$-sheet motifs, along with the other representations purely based on network connectivity, in order to simplify the interpretation of results. Examples of such representation are shown in Supplementary File #1.

HyperBeta was designed to render and analyze MARTINI CG bio-molecular structures, reducing the number of steps required to track crucial structuring phenomena, as usually implemented with VMD and NAMD. Differently from VMD, which mandates the editing of tailored *tcl scripts to analyze and visualize MARTINI CG structures, HyperBeta does not require any additional scripts to analyze these structures. On the contrary, HyperBeta provides an intuitive, interactive and user-friendly interface. In particular, HyperBeta displays a variety of statistics about the identified $\beta$-structures and the dynamic behavior of the system as graphical overlays; this information includes the number, and the type, of CG grains involved in the detected $\beta$-structures. Specifically, HyperBeta detects the identified structures by considering the reciprocal distances and angles formed by CG grains belonging to different peptides, which are used to define the hyper-graph of contacts. Such features will allow the investigation of even larger MARTINI CG-MD outcomes, exploring size and time scales similar to those of laboratory experiments, such as NMR and cryo-TEM. More in details, HyperBeta will allow to elucidate the interplay of inter-molecular interactions completing the experimental theoretical workflow usually adopted for the investigation of self-assembling nanomaterials^25^. We expect that HyperBeta will find immediate applications in the analysis of finer MD trajectories, mapped according to all-atom (AA) and united-atom (UA) model. This will be possible thanks to dedicated executable that allows the mapping and subsequent analysis of protein backbone according to the MARTINI model.

HyperBeta is available for download on GITHUB at the following address: https://github.com/aresio/hyperbeta.

## Methods

In MARTINI CG-MD simulations, peptide self-assembly may lead to $\beta$-sheet rich aggregates, characterized by geometries similar to those of distorted lattices. The method employed by HyperBeta to discover the presence of $\beta$-sheets, deploys the equivalent three-dimensional topological pattern of CG grains. Given a collection of grains represented as a set of points in a three dimensional space, Hyperbeta’s functioning can be summarized into two main steps:

1. all triples of grains belonging to different peptides (hampering the recognition of $\alpha$-helix and random-coil segments), which are “near enough” and “almost aligned”, are found;
2. for each triple, we check which other triples overlap with it, thus suggesting that the grains in these triples belong to the same structure.

In what follows, we formalize these steps and provide an algorithm to discover possible $\beta$-sheets in a collection of grains.

### Basic notions

The L2-norm, or Euclidean norm, of a point $\vec {x} \in \mathbb {R}^3$—in symbols, $||\vec {x}||_2$—is defined as $||\vec {x}||_2 = \sqrt{\sum _{i=1}^3 x_i^2}$ and represents the Euclidean length of the point. Given two points $\vec {x}$ and $\vec {y}$, their distance is the Euclidean norm of their difference, i.e., $||\vec {x} - \vec {y}||_2 = \sqrt{\sum _{i=1}^3 (x_i - y_i)^2}$.

Given three points $\vec {x}, \vec {y}, \vec {z} \in \mathbb {R}^3$, the angle formed by $\vec {x}$ and $\vec {z}$ with respect to $\vec {y}$ is defined as:

$$\begin{aligned} \arccos \left( \sum _{i=1}^3 \vec {v}_i \times \vec {w}_i\right)&\text { with }\vec {v} = \frac{\vec {x} - \vec {y}}{||\vec {x} - \vec {y}||_2} \text { and }\vec {w} = \frac{\vec {z} - \vec {y}}{||\vec {z} - \vec {y}||_2}. \end{aligned}$$

The value of the resulting angle is always between 0 and $\pi$. Notice that the point $\vec {x}$ and $\vec {z}$ always form two angles with respect to $\vec {y}$: either their are both $\pi$, or one of them is acute and one is obtuse. The one that is considered hereby is always the acute one.

### Aligned triples

The concepts of a triple of points (or grains, in our case) pertaining to different peptides that are “near enough” and “almost aligned” is formalized as follows.

#### Definition 1

Given $\alpha \in [0,1]$, $\varepsilon > 0$, and three points $\vec {x}, \vec {y}, \vec {z} \in \mathbb {R}^3$, the ordered triple $(\vec {x}, \vec {y}, \vec {z})$ is defined to be $(\alpha , \varepsilon )$*-aligned* when the following two conditions hold:

- $||\vec {x} - \vec {y}||_2 \le \varepsilon$ and $||\vec {y} - \vec {z}||_2 \le \varepsilon$. That is, the distances between the first and the second point, and between the second and the third point, are both smaller than or equal to $\varepsilon$;
- the angle formed by $\ {x}$ and $\vec {z}$ with respect to $\vec {y}$ is larger than or equal to $\alpha \pi$. Since the angle cannot be greater than $\pi$, it means that the angle must be in $[\alpha \pi , \pi ]$.

**Figure 5:**
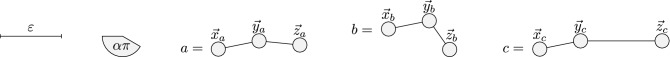
An example of $(\alpha , \varepsilon )$-aligned triples. Let $\alpha$ and $\varepsilon$ be as shown on the left. Then, the triple $a = (\vec {x}_a, \vec {y}_a, \vec {z}_a)$ is $(\alpha ,\varepsilon )$-aligned, the triple $b = (\vec {x}_b, \vec {y}_b, \vec {z}_b)$ is *not* $(\alpha ,\varepsilon )$-aligned (the angle formed by $\vec{x}_b$ and $\vec {z}_b$ with respect to $\vec {y}_b$ is smaller than $\alpha \pi$), and the triple $c = (\vec {x}_c, \vec {y}_c, \vec {z}_c)$ is also *not* $(\alpha ,\varepsilon )$-aligned (the distance between $\vec {y}_c$ and $\vec {z}_c$ is larger than $\varepsilon$).

An example of what are (and what are not) $(\alpha , \varepsilon )$-aligned triples is presented in Fig. 5.

Algorithm 1 shows how finding all $(\alpha ,\varepsilon )$-aligned triples in a set *V* of grains (represented as points in $\mathbb {R}^3$) can be performed. The time complexity of the algorithm is $O(n^3)$, where *n* is the number of grains. The resulting set *T* of $(\alpha ,\varepsilon )$-aligned triples has cardinality bounded above by $n^3$ but, in practical cases, we expect to obtain a set whose cardinality is way lower than this.

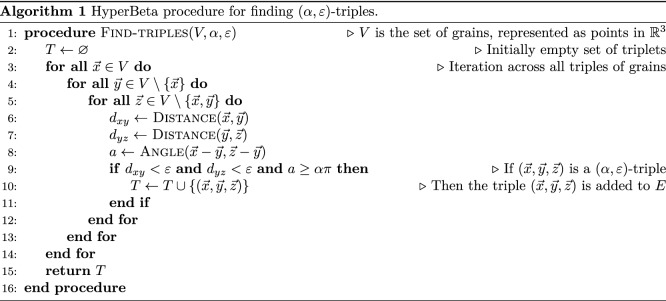



### Connected components

Once all $(\alpha ,\varepsilon )$-aligned triples have been identified, we need to find a way of “gluing” them together if they overlap “enough”, that is, if they share at least two of the three grains.

#### Definition 2

Let $a = (\vec {x}_a,\vec {y}_a,\vec {z}_a)$ and $b = (\vec {x}_b,\vec {y}_b,\vec {z}_b)$ be two $(\alpha ,\varepsilon )$-aligned triples, for some $\alpha \in [0,1]$ and $\varepsilon > 0$. We say that *a* and *b* are *overlapping* when $|\{\vec {x}_a,\vec {y}_a,\vec {z}_a\} \cap \{\vec {x}_b,\vec {y}_b,\vec {z}_b\}| \ge 2$.

**Figure 6:**
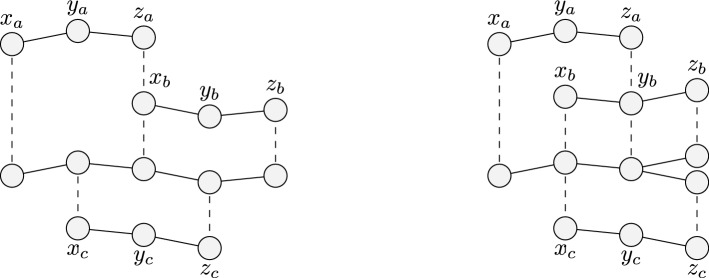
Two examples of structures resulting by “gluing together” a set of overlapping $(\alpha ,\varepsilon )$-aligned triples.

**Figure 7:**
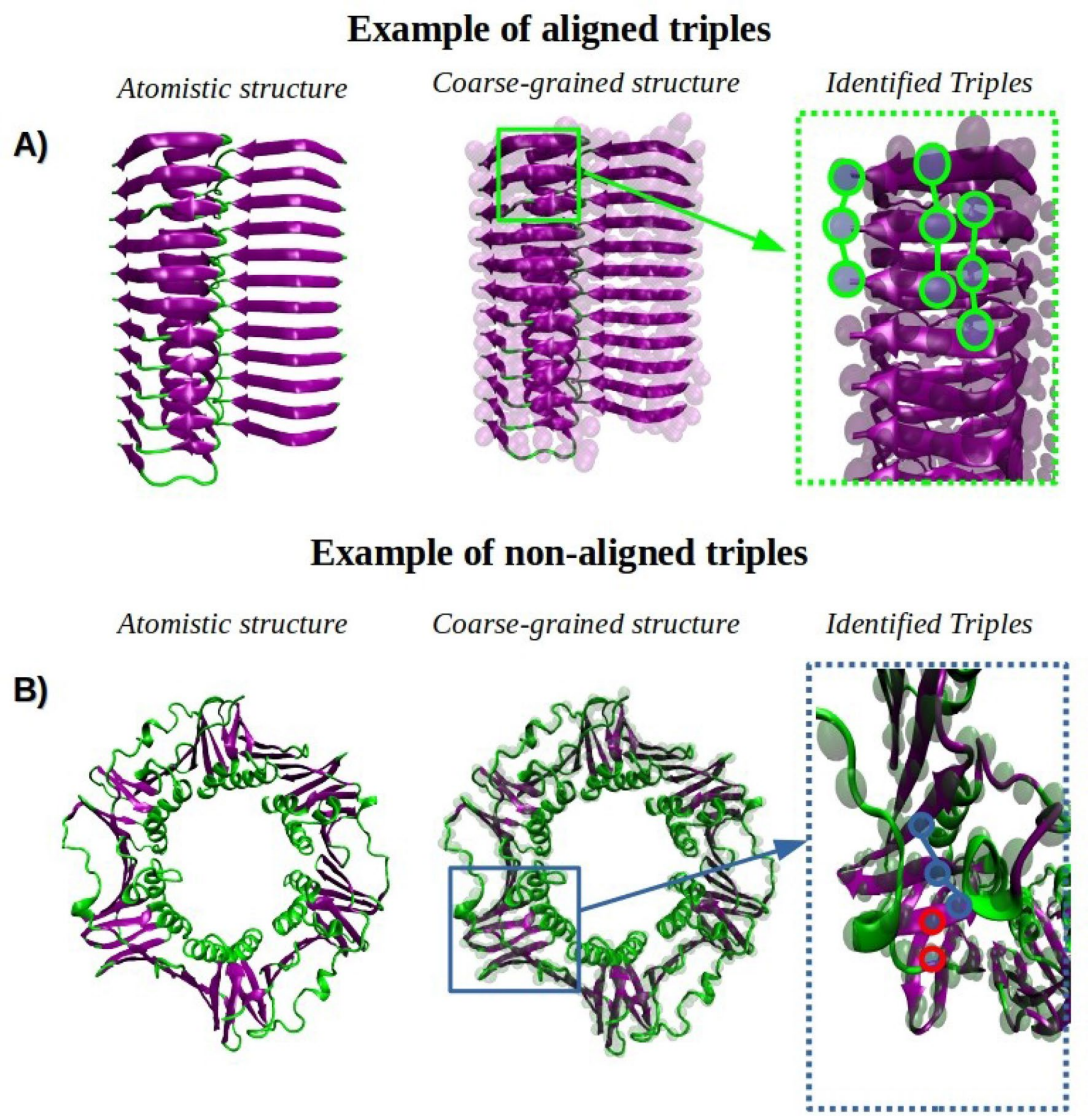
Examples of two real structures showing aligned/not-aligned triples. Green circles: aligned triples. Red circles: grains not belonging to any triples. Blue circles: non-aligned triples. HyperBeta has been validated against different protein structures (see Fig. 2). As shown in panel A, the 42-residue $\beta$-amyloid fibril consists of 12 parallel $\beta$-strands, resulting into aligned triples. Instead, as shown in panel B, the *E. coli* $\beta$-clamp is a ring-shaped homodimer characterized by different secondary structure arrangements. The complex topology, consisting of $\beta$-sheets and $\alpha$-helix domains, hampers the identification of aligned triples.

**Figure 8:**
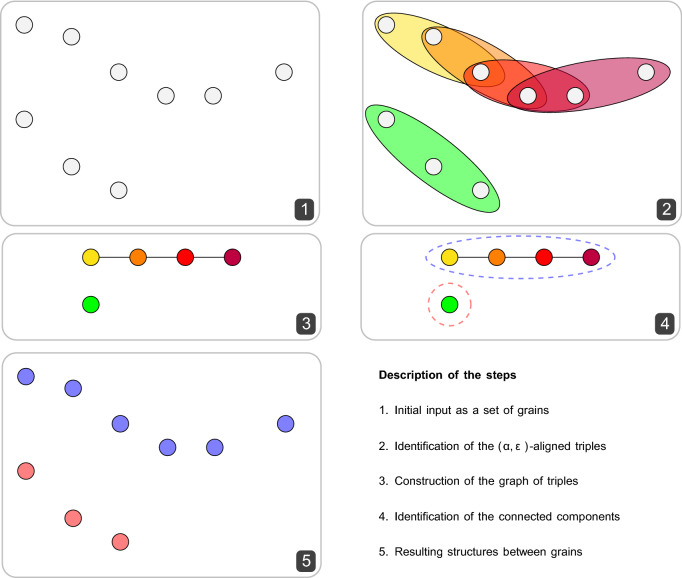
Main steps of the algorithm designed to recognize the presence of $\beta$-sheets.

We can now build a graph *G* where the vertices are the $(\alpha ,\varepsilon )$-aligned triples, and there exists an edge between two vertices if the corresponding triples are overlapping. Notice that the relation is symmetric (i.e., if *a* overlaps with *b* then also *b* overlaps with *a*), thus the resulting graph is undirected. As shown in Fig. 6, overlapping triples can be considered as “pieces” of the same structure once “glued together”. As shown in Fig. 7, the identification of aligned triples allows to easily discriminate the regular alternate $\beta$-sheets domains from $\alpha$-helix domains. When more triples are “glued together” regular $\beta$-sheets are identified. The process of finding which triples can be “glued together” can then be expressed as finding the set of connected components in the graph *G*. The entire process is described by Algorithm 2, and the structure of the resulting algorithm is illustrated with an example in Fig. 8.

The resulting time complexity is given by iterating across all pairs of $(\alpha ,\varepsilon )$-aligned triples, which has quadratic complexity with respect to the number of triples, thus resulting in a time complexity of $O(n^6)$. Depending on the representation employed for the graph, the time needed to compute the connected components is either linear in the number of vertices and edges, or quadratic in the number of vertices, resulting in both cases in a worst-case time complexity of $O(n^6)$. While this time complexity seems high with respect to the number of grains, we remark that this is a *worst-case* scenario and, in practice, we expect the number of $(\alpha ,\varepsilon )$-triples to be much lower than cubic with respect to the number of grains.

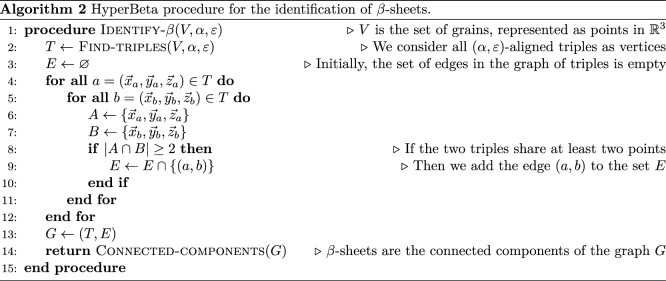



## Supplementary Information

Supplementary Information 1.
