# Supplementary material for: HyperBeta: characterizing the structural dynamics of proteins and self-assembling peptides
Source: Sci Rep. 2021 Apr 8;11:7783. doi: 10.1038/s41598-021-87087-0 (PMC8032683; doi:10.1038/s41598-021-87087-0)
Supplement: Supplementary file 1 — Supplementary Information 1. [file 41598_2021_87087_MOESM1_ESM.pdf]

SUPPLEMENTARY FILE #1

HyperBeta: characterizing the structural dynamics of proteins and self-assembling peptides

Marco S. Nobile[1,5,6,7], Federico Fontana[8], Luca Manzoni[3], Paolo Cazzaniga[4,6,7], Giancarlo Mauri[5,6,7], Gloria A. A. Saracino[2], Daniela Besozzi[5,6,7,+], and Fabrizio Gelain[2,8,+]

[1]Department of Industrial Engineering & Innovation Sciences, Eindhoven University of Technology, The Netherlands

[2]Center for Nanomedicine and Tissue Engineering (CNTE), A.S.S.T. Grande Ospedale Metropolitano Niguarda, Piazza dell'Ospedale Maggiore 3, 20162 Milano, Italy

[3]Department of Mathematics and Geosciences, University of Trieste, Trieste, Italy

[4]Department of Human and Social Sciences, University of Bergamo, Bergamo, Italy

[5]Department of Informatics, Systems and Communication, University of Milano-Bicocca, Milano, Italy

[6]SYSBIO/ISBE.IT Centre for Systems Biology, Milan, Italy

[7]Bicocca Bioinformatics, Biostatistics and Bioimaging Centre (B4), Monza, Italy

[8]Fondazione IRCCS Casa Sollievo della Sofferenza, Unità Ingegneria Tissutale, Viale Cappuccini 1, San Giovanni Rotondo, 71013 Foggia, Italy

[+]Co-corresponding authors.

1 HyperBeta's pre-processing tool

The HyperBeta's pre-processing phase is performed for each snapshot contained in the outcome of Molecular Dynamics (MD) performed by GROMACS. In order to simplify the opening and processing of GROMACS files, HyperBeta provides an intuitive GUI named HyperBeta Processing Tool (HPT). Figure 1 shows a screenshot of HPT's main window, highlighting its six parts. The main button (1) opens the secondary window for GROMACS file import and

processing (Figure 2). By using this window it is possible to select an input GROMACS file (button 8). HPT will automatically identify and split multiple MD snapshots contained in the GROMACS file. In order to properly identify and process the peptidic information contained in the MD snapshots, the user must specify three values: the number of amino-acids contained in each peptide (10); the angular threshold α (11); the distance threshold ε (12), measured in ångströms (Å). The user must also specify the interval of indexes of the snapshots to be processed $[s, t)$ (13), and whether any already processed snapshots must be skipped or be processed *ex novo* (9). Once everything is set, the algorithm for β-sheets identification can be launched by pressing the Process button (14). The algorithm will create a sequence of sub-folders named "snapshotXX", where XX $\in [s, t)$.

As soon as the requested snapshots are processed, the β-structures can be visualized. This step is performed by opening the output folder using the Browse button (2) in the main window (Figure 1). The default GRO files prefix of sub-folders is automatically set to "snapshot", although it can be changed by using the dedicated text area (3). Finally, the user can specify the interval of MD snapshots to be visualized (4) and launch the HyperBeta Visualization Tool (HVT) by pressing the Run button (6). Please note that HVT must be compiled separately. The path to the compiler can be specified using the button (5); if the executable file is correct, HyperBeta will show a confirmation message in the field (7).

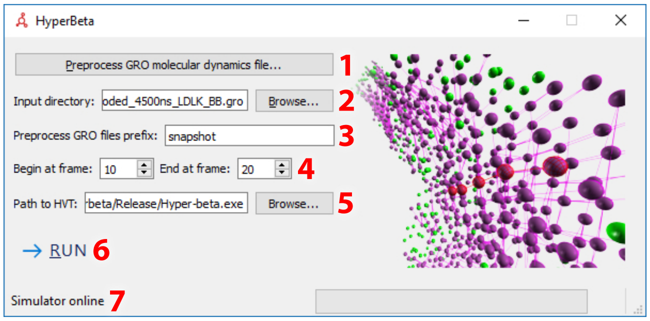

Figure 1: HyperBeta Processing Tool, main window.

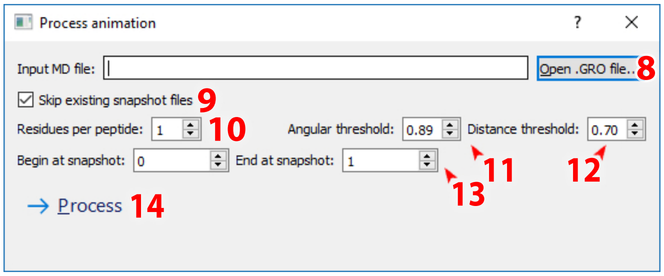

Figure 2: HyperBeta Processing Tool, processing window.

2 HyperBeta's visualization tool

Once HyperBeta's pre-processing is completed, the outcome can be visualized by HyperBeta's visualization tool. HVT is a cross-platform real-time rendering engine that was implemented using C++, OpenGL, FreeGLUT and the GLEW extensions.

HVT displays the grains using the whole window (or screen, when full-screen mode is activated). On the left side of the window, a translucent toolbar—which can be hidden by pressing the key "Q"—displays some data about the structure under analysis (top) and contains some service buttons (bottom). The functionalities provided by the toolbar will be described later in this section.

HVT displays the grains of the MD as solid spheres. When set in *default mode*, the grains appear as green spheres. The grains involved in subsets corresponding to β-structures appear as purple spheres, and are connected by purple bars.

HVT provides multiple selection and inspection modes:

- *grain selection mode*: a grain can be selected by clicking on it with the left mouse button. When a grain is selected, it is highlighted with a rotating wire-frame golden sphere. Grain selection mode also displays the type of amino-acid, and the ordinal number of the peptide it belongs to;
- *peptide selection mode*: by activating the grain selection mode and pressing the key "F3", the peptide selection mode is activated. In this modality, the whole peptide containing the selected grain is highlighted with partially translucent spheres;
- *structure selection mode*: a single β-sheet identified by HyperBeta can be highlighted by clicking with the right mouse button. When a β-sheet is selected, it is highlighted with rotating wire-frame red spheres. The connecting bars of such structure are now colored in red;
- *structure emphasis mode*: all β-sheets are rendered with a translucent purple surface, except the selected β-sheet. This mode, which emphasizes the selected structure, can be enabled by pressing the key "O";
- *rainbow mode*: HVT automatically renders each peptide using a unique color. This mode can be enabled by pressing the key "B".

Figure 3 shows an example of rendering when all the selection modes are active.

The user can inspect the β-sheets by navigating through the nano-space. Navigation is performed using the keyboard. The keys associated to camera movements and structures rotation are listed in Table 1.

In order to support the visualization of large-scale structures, HVT leverages display lists and dynamically performs back-face culling, aggressive frustum culling, and Level-of-Detail (LoD) balancing, that is, the number of polygons used to represent the grains is inversely proportional to their distance from the virtual camera. LoD can be toggled by pressing the key "L".

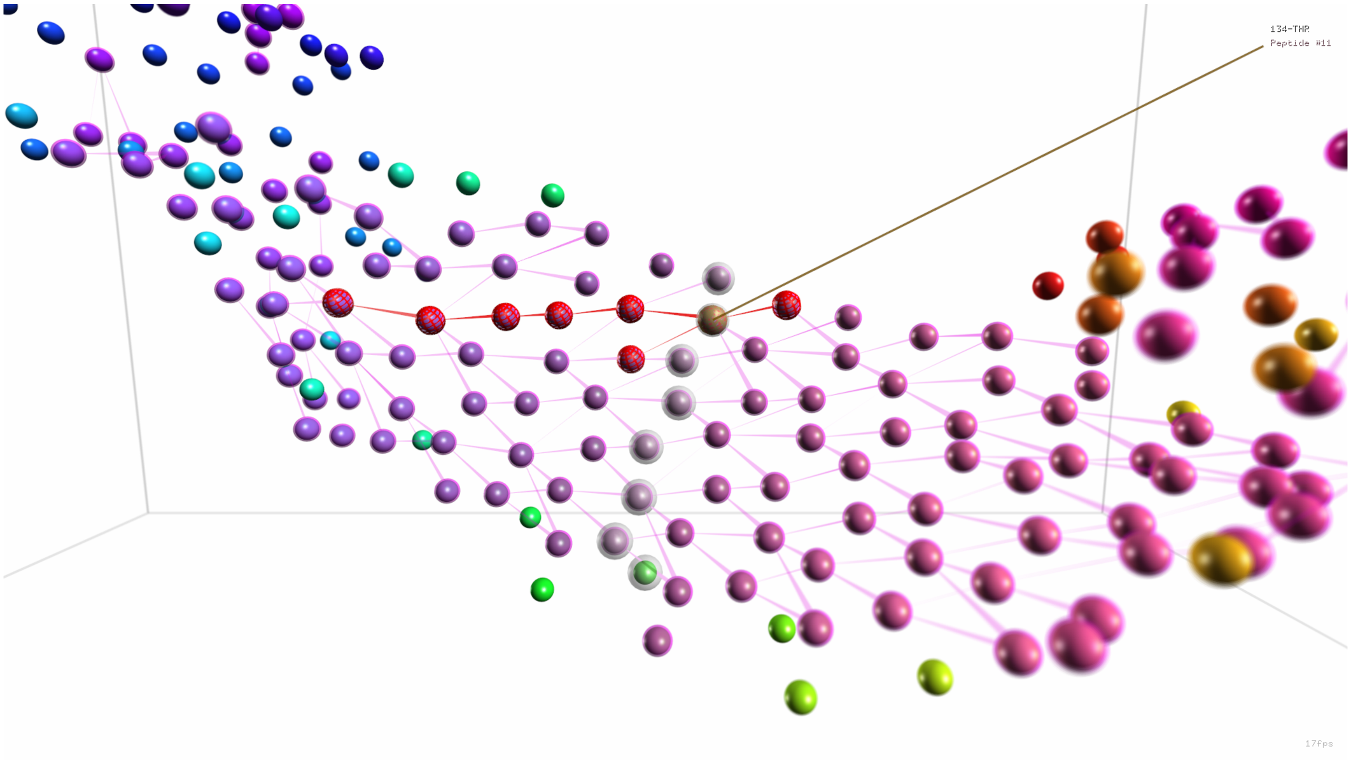

Figure 3: Example of selection modes. A grain of type 134-THP is selected, as denoted by the golden wire-frame sphere. The grain belongs to the 11-th peptide in the GROMACS file, denoted by the translucent spheres. The β-sheet involving the grain is denoted by red wire-frame spheres. The structure emphasis mode is enabled, so that the non-selected β-sheets are represented using translucent purple spheres. Finally, the rainbow mode is enabled, so that all peptides are represented using unique colors.

HVT was specifically designed to simplify the interpretation of peptidic aggregates. Thus, the renderer implements advanced photographic concepts, like diaphragm aperture, which is used to simulate depth-of-field blurring (DoF) of out-of-focus grains. DoF is exploited to provide visual clues of depth relationships between grains [1]. Figure 4 shows a comparison of the same region of the structure, when the user sets the focus on different grains by clicking on a grain with the left button. The DoF effect is calculated by accumulating 24 images from jittered positions through the lens into the Accumulation Buffer [2]. The opening of the diaphragm and the focal length can be changed at real-time, in order to simplify the analysis of the nano-structures. DoF simulation can be computationally expensive [2], affecting the reactivity of the renderer. Thus, in order to ensure a pleasant experience to the user, HVT automatically decreases the number of samples to 8 when the camera, or the grains, are moving on the screen.

In order to provide additional visual clues about the relative spatial positioning of grains, HVT supports motion blur, fogging, dynamic lighting, and dynamic shading. To ensure high-quality alpha blending, HVT also supports optimized depth sorted primitives rendering [3]. All these features can be toggled by using the keys listed in Table 2, or by using the icons in the lower corner of the toolbar.

One further functionality offered by HVT is the visualization of animations. When the GROMACS file contains multiple snapshots, HyperBeta can process all frames of the MD, calculate the β-sheets for the peptidic structures in that frame, and visualize the assembly process as an animation. To this aim, HVT provides an additional set of icons

Table 1: Navigation keys.

Function	Key
Move camera forward / backward	W / S
Strafe camera on the left / right	A / D
Rotate camera on the left / right (yaw)	Left / Right
Tilt camera up / down (pitch)	Up / Down
Rotate camera counterclockwise / clockwise (roll)	Page Up / Page Down
Reset position of camera	r
Control rotation of the structure around the x axis	X (shift-x)
Control rotation of the structure around the y axis	Y (shift-y)
Control rotation of the structure around the z axis	Z (shift-z)
Halt the rotation of the structure around all axis	Space

Table 2: Renderer keys.

Function	Key
Toggle sounds	F1
Toggle fogging	F2
Toggle peptide selection mode	F3
Toggle depth-of-field simulation	F4
Toggle full screen	F5
Toggle display non-beta grains	F6
Toggle display β-structures	F7
Toggle bounding box	F8
Toggle visualization of β-sheets	T
Toggle dark background	C
Toggle time inversion	I
Toggle grains statistics	U
Toggle LoD balancing	L
Toggle toolbar	Q
Increase the opening of diaphragm	K
Decrease the opening of diaphragm	J
Reset the opening of diaphragm to default	F

in the lower side of the toolbar, whose semantics is similar to those of a common media player.

HVT also supports a more classic visualization of β-sheets motifs as shown in Figure 5. The spatial information of the motifs is pre-calculated by HyperBeta, on the fly, during the hyper-graph generation. This representation can be engaged and disengaged by pressing the "T" key.

Finally, HVT supports the calculation of statistics of the structures calculated on all snapshots of the MD. Specifically, HVT displays some histograms of the number of connected β-structures, the number of triples, and the ratio of grains involved in β-structures. HVT can also display the frequency of peptides involved in β-sheets, in each snapshot. This information, hidden by default, can be shown as an overlay next to the button bar by pressing the key "U" (see Figure 6 for an example).

Finally, HyperBeta supports dark background (key "C"), full-screen rendering (function key "F5"), and can hide all panels (key "Q") and statistics (key "U"), as shown in Figure 7.

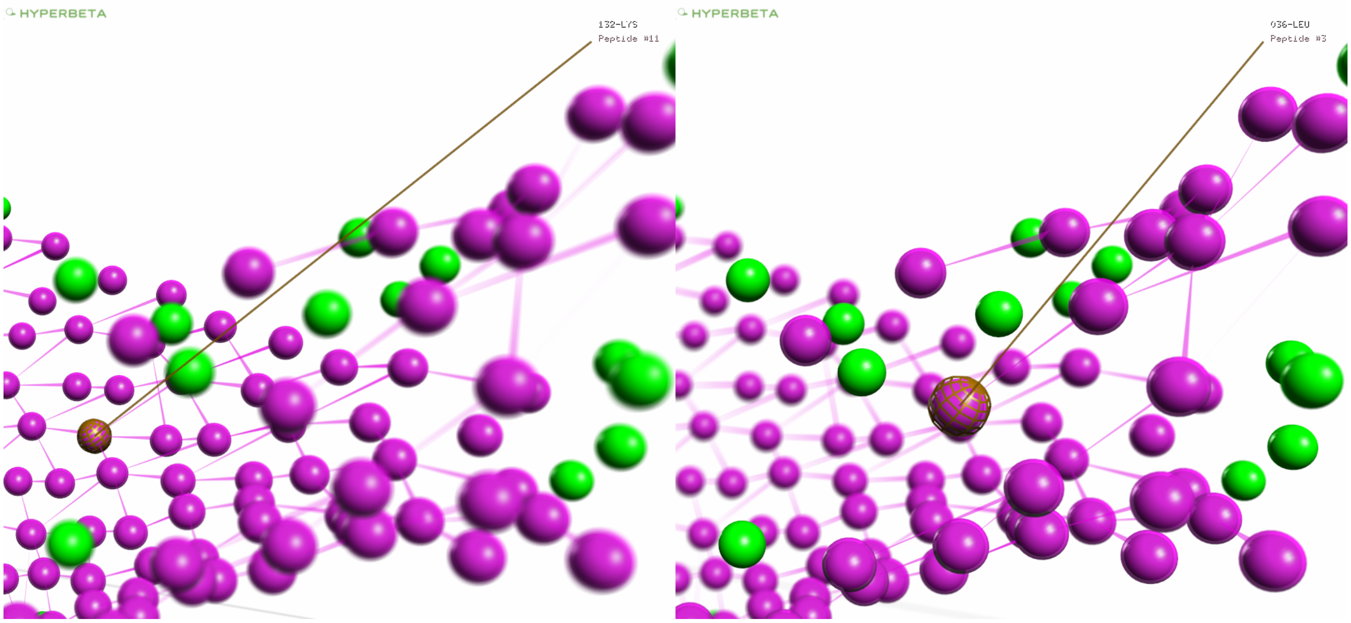

Figure 4: Selection of grains and simulation of depth-of-field blurring. Both the parallactic distortion and DoF simulation provide visual cues about the spatial relationships of the grains.

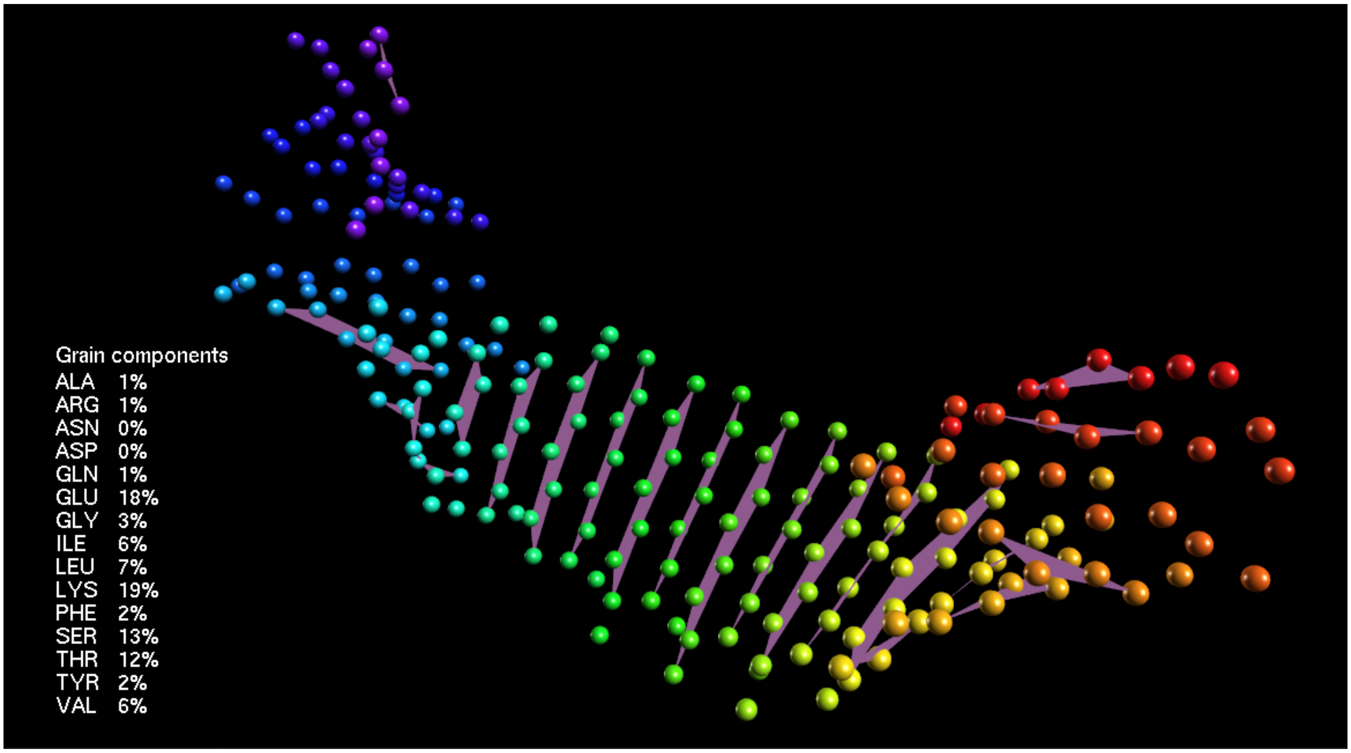

Figure 5: Beta-sheet motifs rendered by HVT.

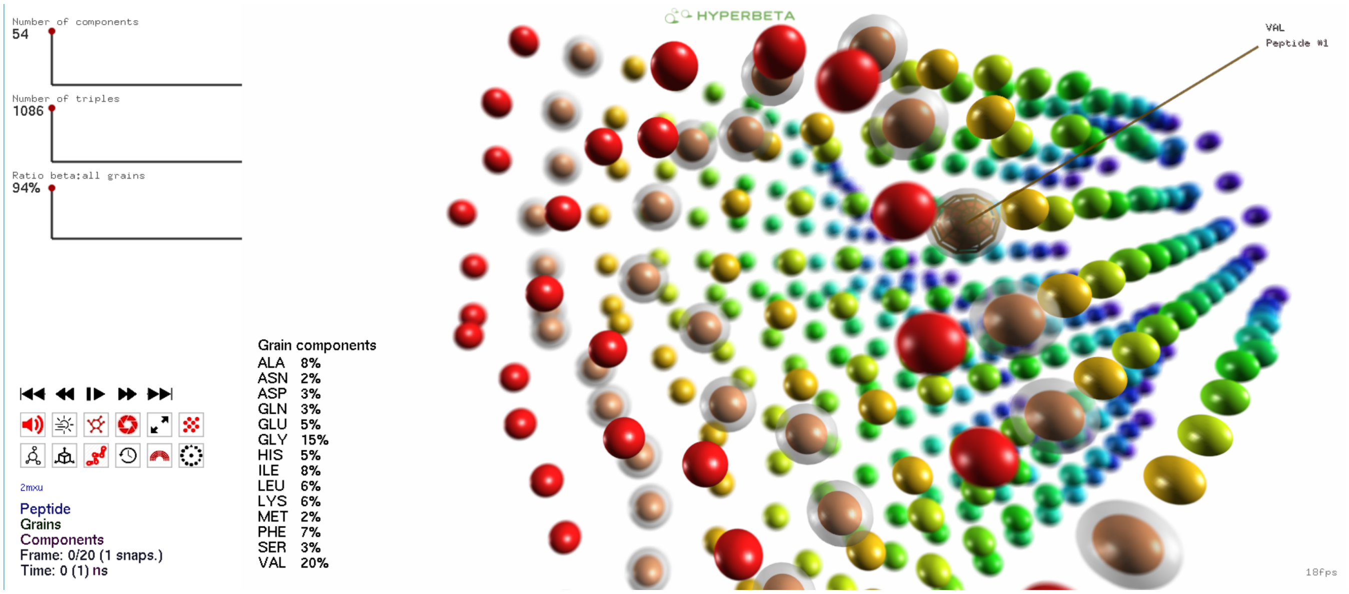

Figure 6: Example of statistics.

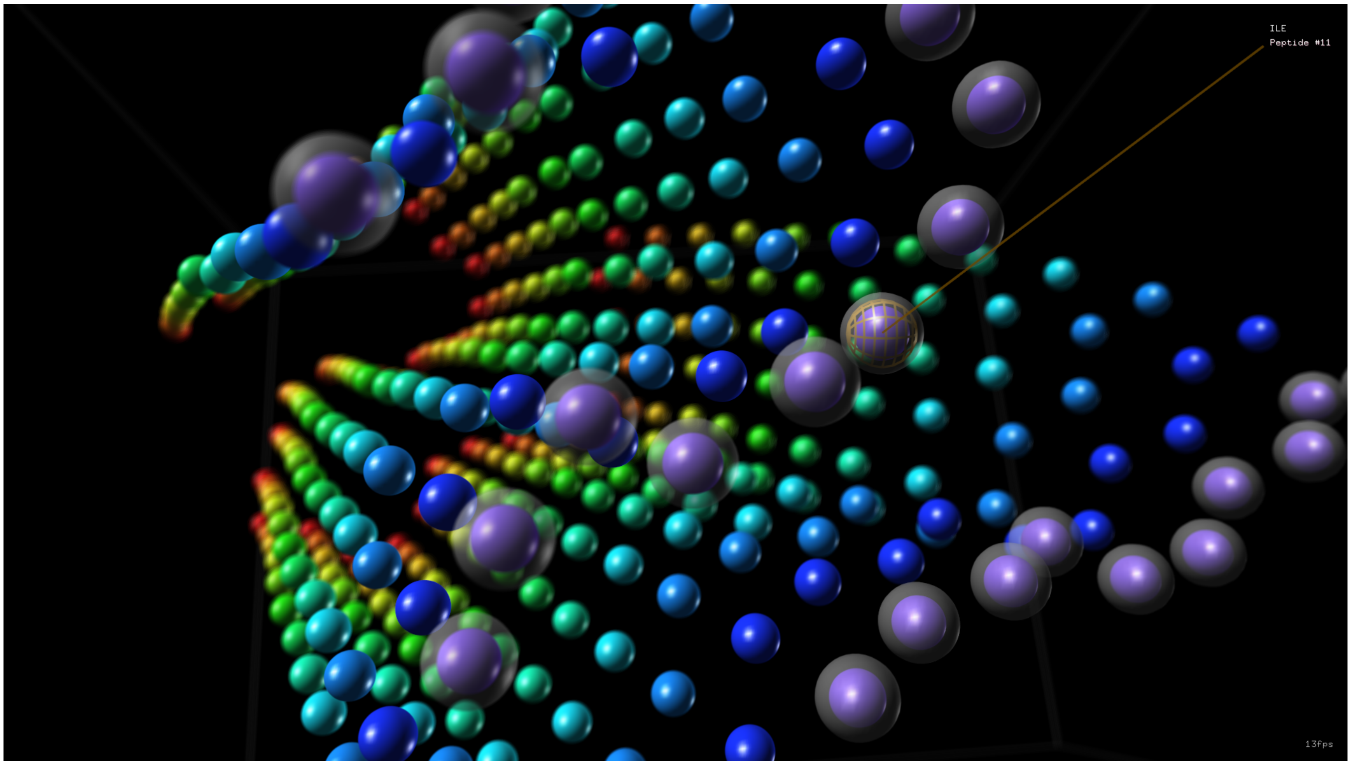

Figure 7: Full-screen, panels-free and dark background rendering with HyperBeta.

3 Parameter sweep analysis on the parameters α and ε

In order to assess the impact of the parameters α and ε on the identification of β-sheets, we performed a parameter sweep analysis (PSA) on their values. Specifically, we varied the two parameters on a regular lattice in the intervals $\alpha \in [0.5, 1.0]$, $\varepsilon \in [0.39, 0.99]$, and we tested 31×31 combinations of these values on the structures 2mxu, 2fkg and 3bep (presented in the main text), which are known to contain β-sheets, and the structure PSMα3, not including any β-sheet.

The results of the PSA are shown in Figure 8. In all panels, we show the value of α on the x axis, the value of ε on the y axis, and the number of grains assigned to β-sheets on the z axis. To interpret the results reported in the plots, we use as reference the number of grains in β-sheets estimated by STRIDE, whose value corresponds to the bright area. Conversely, the purple areas denote an over-estimation of the number of grains with respect to STRIDE, while the orange areas denotes an under-estimation. The red star on the surface indicates the number of grains estimated using HyperBeta's default setting, that is, $\alpha = 0.89$ and $\varepsilon = 0.7$nm. Our results show that, in the case of 2fkg and 3bep, HyperBeta's prediction agrees with the number of grains in β-structures returned by STRIDE, since the star lies in the bright area. In the case of 2mxu, HyperBeta seems to over-estimate the number of grains belonging to β-sheets. However, such difference is ascribable to the strong β-strands alignment, as highlighted through ssNMR analsysis [4]. STRIDE can underestimate the number of β-structures, because it relies on calculating the energies of hydrogen bonds, which can be slightly distorted.

Finally, HyperBeta correctly recognizes a structure completely lacking any β-sheets, as shown by the result on the PSMα3, which is a highly toxic 22-residue phenol-soluble modulin α3 peptide secreted by *Staphylococcus aureus* [5]. According to our results, HyperBeta correctly returns the absence of β-sheets, except when "extreme" settings are used (e.g., $\alpha = 0.5, \varepsilon = 1.0$nm). Overall, the outcome of the PSA confirms the accuracy and robustness of our method.

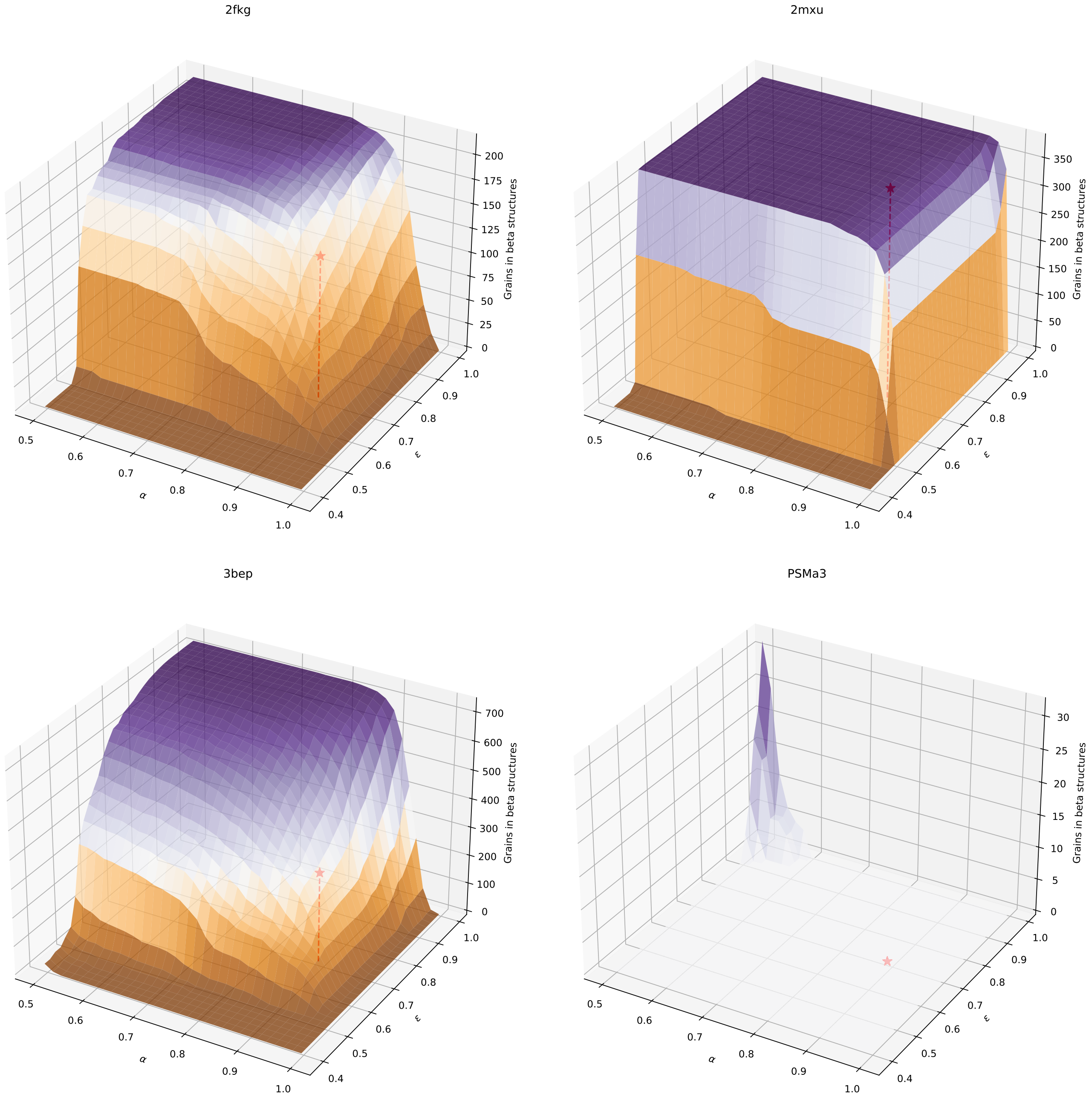

Figure 8: Results of the PSA on the structures 2fkg, 2mxu, 3bep and PSMα3.

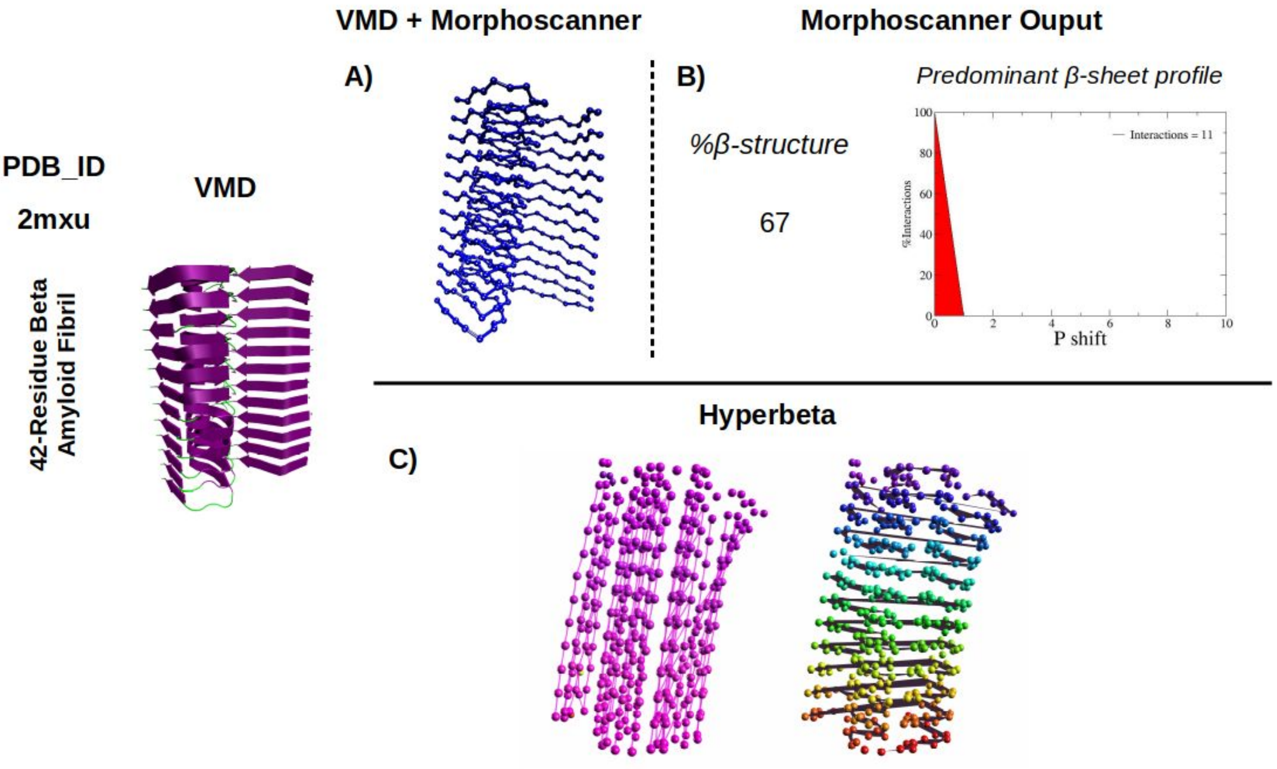

Figure 9: Comparison between HyperBeta and Morphoscanner analysis workflow. The reference structure (the 42-residue amyloid fibril, PDB ID: 2mxu) is presented using VMD. The same structure was analyzed with Morphoscanner and HyperBeta. A) The CG structure is visualized through VMD, highlighting the β-sheets identified by Morphoscanner. B) Morphoscanner returns the percentage of grains belonging to β-sheet structures and the predominant β-sheet profile, quantifying the strands displacement in the CG structure. C) The CG structure is analyzed and visualized using HyperBeta. In addition to the multiple statistics (see Fig. 6), HyperBeta can be used for the visualization of CG structure, highlighting the β-sheet structures.

References

[1] D.A. Atchison and G. Smith. *Optics of the human eye.* Butterworth-Heinemann, 2000.

[2] J. Demers. Depth of field: A survey of techniques. In *GPU Gems 3*, chapter 23. Addison-Wesley Professional, first edition, 2007.

[3] M. Maule, J.L.D. Comba, R.P. Torchelsen, and R. Bastos. A survey of raster-based transparency techniques. *Computers & Graphics*, 35(6):1023–1034, 2011.

[4] Y. Xiao, B. Ma, D. McElheny, S. Parthasarathy, F. Long, M. Hoshi, R. Nussinov, and Y. Ishii. Aβ (1–42) fibril structure illuminates self-recognition and replication of amyloid in Alzheimer's disease. *Nature structural & molecular biology*, 22(6):499, 2015.

[5] Einav Tayeb-Fligelman, Orly Tabachnikov, Asher Moshe, Orit Goldshmidt-Tran, Michael R Sawaya, Nicolas Coquelle, Jacques-Philippe Colletier, and Meytal Landau. The cytotoxic Staphylococcus aureus PSMα3 reveals a cross-α amyloid-like fibril. *Science*, 355(6327):831–833, 2017.
